# Assessment of pattern and treatment outcome of patients admitted to pediatric intensive care unit, Ayder Referral Hospital, Tigray, Ethiopia, 2015

**DOI:** 10.1186/s13104-018-3432-4

**Published:** 2018-05-24

**Authors:** Hansa Haftu, Tedrose Hailu, Araya Medhaniye, Teklit G/tsadik

**Affiliations:** 1 Department of Pediatrics and Child Health, College of Health Sciences, Mekelle University, Mekelle, Ethiopia; 2 Mekelle University, Mekelle, Ethiopia

**Keywords:** PICU, Mortality, Pattern, Inotropes

## Abstract

**Objective:**

To describe admission pattern and outcome with its predictor variable on the mortality of children admitted to pediatric intensive care unit (PICU), Ayder Referral Hospital, Northern Ethiopia, from September 2012 to August 2014.

**Result:**

From 680 admitted patients, 400 patients were analyzed. Average age at admission was 62.99 ± 60.94 months, with F:M ratio of 1:1.2. Overall (from infectious and non-infectious) the most commonly affected systems were respiratory (90/400 pts., 22.5%) and central nervous system (83/400 pts., 20.75%). Most were admitted due to meningitis (44/400 pts., 11%), post-operative (43/400 pts., 10.8%) and acute glomerulonephritis (41/400 pts., 10.3%). The overall mortality rate was 8.5%. Multivariable logistic regression shows, use of inotropes (p = 0.000), need for mechanical ventilator (p = 0.007) and presence of comorbid illness (p = 0.002), infectious cause (p = 0.015) and low level of Glasgow coma scale less than eight (p = 0.04) were independent predictors of mortality. From this study, common cause of PICU admission and death was meningitis. This highlights the importance of focusing on the preventable methods in the public such as vaccine, creating awareness about hygiene, and expanding ICU for early detection and for treatment acutely ill children.

**Electronic supplementary material:**

The online version of this article (10.1186/s13104-018-3432-4) contains supplementary material, which is available to authorized users.

## Introduction

An intensive care unit (ICU) is a specially staffed and equipped, separated area in a hospital, dedicated to the management of patients with life-threatening illnesses [1]. According to World Health Organization (WHO), the major causes of death in under-five children in developing countries are preventable and curable diseases, if the care is optimized [2, 3]. The majority (99%) of childhood deaths occurring in developing countries, especially under-five mortality, is highest in sub-Saharan Africa (> tenfold) [4, 5]. Intensive care could reduce mortality rates by 15–60%, when it is a well-equipped, and staffed with intensivists [2, 6].

There was a study conducted in Jimma, Ethiopia, mortality was 40% with the most common cause of admission and death being trauma [6]. A similar study of critical care unit of all age patients in Tanzania revealed mortality rate of 41.4% [6]. In a study of severe head injury patients in the ICU of National Hospital Abuja in Nigeria, the mortality rate was 68.4%, [7, 8].

In sub-Saharan Africa, ICUs have varying qualities and quantities of infrastructure necessary for the provision of proper critical cares services. The reported disease characteristics and mortality rates of patients admitted to ICUs in sub-Saharan Africa vary widely from one population to another. The regional hospitals send their critical patients to these referral hospitals for ICU care. Demographic profile and outcome of PICU patients can vary widely in different studies while there is a scarcity of data in Ethiopia’s critically ill children. The aim of the present study was to describe the demographic profile and the outcome of our PICU patients, to evaluate the relationship of the outcome to diagnostic categories and treatment characteristics, and to investigate mortality risk with possible outcome predictors.

## Main text

### Methods

Ayder Referral Hospital (ARH) is the largest referral in Northern Ethiopia. It started as a referral and specialized medical center in 2008, providing service to about 8 million people. A study was conducted in nine bedded PICU from 1st to 25th July 2015. This data was obtained from ICU logbook and patient charts. A retrospective cross-sectional study design was used.

### Sample size and sampling method

All consecutively admitted patients to PICU from September 2012 to August 2014 were included, based on the criteria. *Inclusion and exclusion criteria*: all patients admitted to PICU whose age is 14 days–18 years were included in the study. The cutoff age was determined to be 14 days because after 1 year of study, those critically ill patients in the age of 7–14 days were admitted to neonatal intensive care unit, so that the exact pattern of diagnosis and age distribution of these patients would be incomplete. Patients with incomplete or missed data were excluded from the study. Other patients excluded from the study were, those who died on arrival (within 2 h of admission); this is not sufficient time to give optimal care in the ICU, and because the outcome of these patients is related to the emergency or other ward care.

### Study variable

#### Dependent variable

Outcome.

#### Independent variable

Age, sex, length of ICU stay, diagnosis at admission, need for a mechanical ventilator, length of mechanical ventilation stay, inotropes use, comorbid illness, disease character (medical vs. surgical and infectious vs. non-infectious), admission sources, Glasgow Coma Scale (GCS) at admission.

### Data collection

The following data was collected retrospectively: age, gender, admission diagnosis, presence of comorbidities, admission sources, treatment characteristics; the need for mechanical ventilation (MV) and MV days, the need for inotropes, length of stay (LOS), outcome and cause of death. Neurologic status was evaluated using the pediatric version of GCS. The questionnaire was adapted by reviewing different literature. Two medical interns were employed in the data collection process and one general practitioner as a supervisor. A 2 days training was given to them on the objectives of the study, the contents of the questionnaire and on issues related to the confidentiality. Five days prior to the data collection, a pre-test was conducted in Mekelle Hospital in 2.5% of the sample size for completeness of the data collection format. Based on the findings of the pre-test, some questions were modified and some others were added for estimating wealth index. The principal investigator was continuously supervising the data collectors for completeness and consistency and the records were cross-checked.

### Data analysis

Data was cleaned, edited and entered into Epi data version 3.1 and analyzed using SPSS software (version 16.0). Characteristics of study participants were analyzed using descriptive statistics. After multicollinearity was checked using IF < 10 and Tolerance test > 0.1, variables having *p* value ≤ 0.25 at bivariate logistic regression analysis were fitted into multivariable logistic regression. Bivariate and multivariate logistic regression was used to identify the association between dependent and independent variables. Multiple logistic regressions with a calculation of adjusted odds ratios were used to determine the influence of covariant on mortality. Statistical significance was considered at a p value of < 0.05. Odds ratio with 95% confidence interval was used to show the strength of association between independent and dependent variables.

### Results

Among 680 consecutive patients admitted in the study period, 400 patients were analyzed. Of the total admitted patients, 215 (53.8%) were male and 185 (46.2%) were female, giving a male: female ratio of 1.2:1. The average age of admission was 62.99 ± 60.94 months. From the study participants, 250 (62.5%) were under five (Table 1). A majority were admitted from EOPD (288/400 pts., 72%), a transfer from ward (65/400 pts., 16.3%).

**Table 1:** Sex and age wise distribution of the study group

Variable	Frequency	Percentage
1. Age (years)		
Less than 1	101	25.3
1–2	62	15.5
2–5	87	21.8
5–11	76	19.0
11–18	74	18.5
2. Sex		
Female	185	46.2
Male	215	53.8

**Fig. 1:**
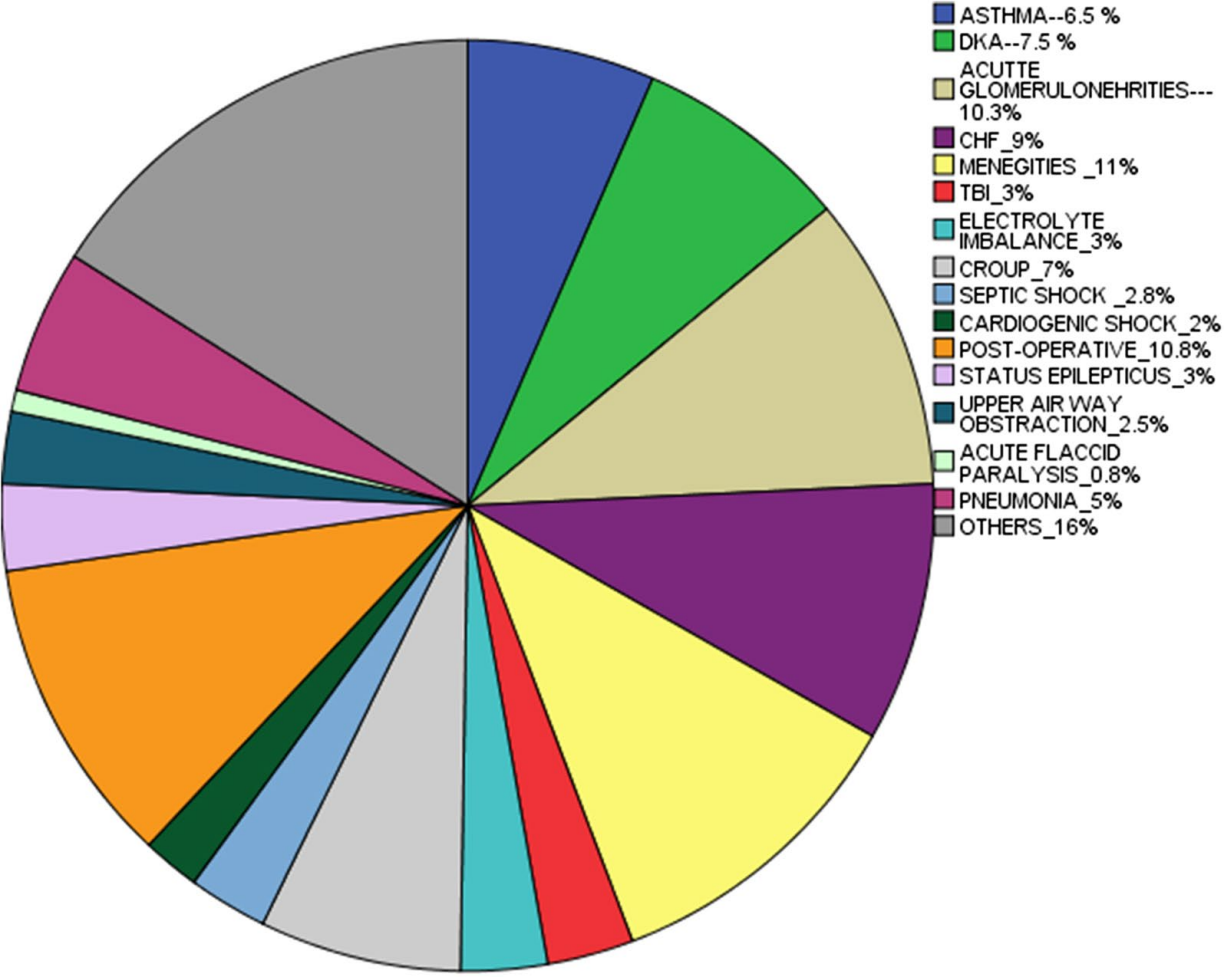
Common causes of ICU admission by their diagnosis. N.B—others-malignancy, poisoning, near-drowning, disseminated infection, brain tumor and alcohol intoxication

The mean ICU stay was 4.9 ± 5.8 days (range 1–30 days) with a majority (61%) of them staying for 2–7 days. The vast majority of patients admitted to ICU were due to medical problems (85.2%) and non-infectious disease (266/400 pts., 66.5%) (Table 2). Most were admitted due to meningitis (44/400 pts., 11%), post-operative (43/400 pts., 10.8%) (Fig. 1).

**Table 2:** System wise distribution of the patients admitted in PICU

Variable	Frequency	Percentage
1. Non-infectious (N = 266)		
Cardiac	55	20.7
Renal	47	17.7
Respiratory	38	14.3
CNS	38	14.3
Endocrine	31	11.7
GIT	29	11
Trauma	14	5.3
Others*1	29	10.9
2. Infectious (N = 134)		
Respiratory	52	38.9
CNS	45	33.6
Sepsis	27	20.7
Others*2	6	4.4
3. Characteristics of the disease at initial ICU admission		
Medical	341	85.2
Surgical	37	9.3
Both	22	5.5
4. Characteristics of the disease at initial ICU admission		
Infectious	134	33.5
Non-infectious	266	66.5

N.B: *1-poisoning, drawing, alcohol intoxication, fluid and electrolyte imbalances, hematology

*2-renal, musculoskeletal system, malaria

Thirty-four patients died, given a mortality rate of 8.5% and the immediate cause of death in most patients was multi-organ failure (MOF) (42.9%) (Additional file 1: Table S1).

Mortality analysis in relation to different diseases (Additional file 1: Table S2) shows, meningitis (8/34 pts., 23.6%), cardiogenic shock (7/34 pts., 20.6%) followed by pneumonia (3/34, 8.8%) were the major causes of death in this study. Most patients admitted with pneumonia (18/36 pts., 90%) and croup (25/28 pts., 89.35%) were under five (Additional file 1: Table S3). Study participants with comorbid illness account 45.8% (183/400 pts.) with a majority of them having one comorbid (41%, 75/183 pts.). Mortality was higher in those patients with comorbid illness, 14.8% vs. 3.2%, which is statistically significant. From study participants, 16 (4%) of patients were a candidate for MV, and 3.5% of them were intubated with 35.7% died. Even though the length of MV was not associated with increased rate of mortality, length of ICU stay was an independent predictor of mortality. Thirty patients (7.5%) were on inotropes, 56.7% (17/30) of patients survived and 43.3% (13/30 pts.) died. Age and gender were not statistically significant risk factors for mortality. Multivariate logistic regression was used to calculate the association between variables. Patients who had comorbid illnesses were 10 times more likely to die than those without [AOR = 10.2 (2.4–44), CI = 95%]. There was strong association between the level of consciousness and mortality. As a result of severe impairment of consciousness (GCS < 8) increase mortality by 7.8 times as compared with those mild level of impaired consciousness [AOR = 7.75 (1.1–54), CI = 95%]. Patients who needed MV were at 17.6 times increased risk of mortality than those who did not need MV [AOR = 17.6 (2.2–14.3), CI = 95%]. Regarding treatment with inotropes, patients who needed inotropes were at increased risk of mortality by 10 times than those who did not need inotropes [AOR = 10.4 (3.7–29), CI = 95%]. Mortality was high in those patients who had an infectious diagnosis than non-infectious and long ICU stay than short ICU stay, which was statistically significant. With regard to the admission diagnosis, case fatality was high in those patients with septic shock and acute flaccid paralysis (Additional file 1: Table S4).

### Discussion

The purpose of this study was to assess pattern and treatment outcome, and its predictors of children admitted to PICU, in ARH. Mean age of the admitted patient was (62.99 ± 60.94) month, with 62.5% of them under 5 years and M: F ratio was 1.2:1. The mean ages of admitted patients were higher in our ICU than studies done in Greece (54.26 ± 49.93 months) and India (40.01 ± 45.79 months [9, 10]. This may be due to the wide age (2 weeks–18 years) distribution of patients admitted to our ICU. However, the age distribution of ICU admission of which, the majority were under five (62.5%), was similar to studies done in India, 53–72.4% were under five [9–13]. The preponderance of male sex (53.8%) was similar to study done in Ethiopia − 54.7% [6] and Brazil − 55.2% [13], but somehow lower than studies done in India, Nepal, and Greek (54–61.1%) [9–12, 14].

The majority of admitted patients were medical (85.3%) with the commonly affected system, respiratory (90/400 pts., 22.5%) and CNS (83/400 pts., 20.75%). The demographic profile of our patient was similar to studies done in Greek, of which major cause of admission was due to pathologic emergencies (69.8%) and respiratory system (22.3%) involvement [10]. This was opposite to studies done in Jimma, Ethiopia, and Tanzania where surgical and trauma patients represent a large proportion of PICU patients [6, 7, 15]. This difference may be due to common ICU for all cases and all age groups.

However, the common cause of under-five ICU admission was infectious (79.1%) with the most common being pneumonia, septic shock, meningitis and croup which was similar to studies done in Ethiopia and Tanzania [3, 4, 6, 7]. Thirty-four patients died giving a mortality rate of 8.5%. The reported mortality rate varies from 2.1 to 41% [6, 7, 9, 10, 12–16] with the highest mortality in developing countries due to lack of resources [6, 7]. Even though mortality in our patients was within the reference range, it’s relatively high compared to recent studies done in India [9] and China [14], that was 2.1 and 6.5% respectively. This is related to inadequate resources similar to other developing countries [16]. However, this was lower than studies done in other sub-Saharan countries (40–42%) [6, 7, 17, 18]. This may be due to the presence of isolated PICU (others use common ICU for all age) and burn unit of which burns contribute to high case fatality rate for those who have common ICU for all cases [7].

Patients with comorbid illness were at higher risk of mortality (p = 0.002) than those without (14.8% vs. 3.2%), which is similar to studies done in Nepal and China [12, 14, 16].

### Conclusion

The mortality rate of our PICU was 8.5%, with the most common cause of admission and death being infectious causes mostly affecting respiratory and CNS. The statistically significant predictors of mortality in this study were: the presence of comorbid illness, need for MV, need for inotropes, low GCS level, infectious disease and duration of ICU stay. The need for ventilation and inotropes indicates that these patients were in an advanced stage of a disease (Additional file 2).

### Recommendation

The Federal Ministry of Health and Regional Health bureaus in collaboration with ARH should invest on creating awareness in the community on the preventing mechanism, such as, improving vaccine coverage, hygiene, and educating the population about the disease so that they will get treatment in the early stages of a disease. The regional health bureaus must also consider expanding PICU in the region to address critically ill patients.

Ayder Referral Hospital has also to work more on PICU providing a high-quality intensive care to critically ill patients focusing on increase ICU capacity.

## Limitation of the study

The limitations of this study include the retrospective design; the mortality rate may be falsely low in the presence of limited resources, due to a significant number of patients were not analyzed because their data was incomplete. This provides quantitative data in the available resources, but not on the specific quality of care delivered.

## Additional files

**Additional file 1: Table S1.** Patients’ outcome at the end of ICU stay. **Table S2.** Mortality across diagnostic categories (N = 34). **Table S3.** Admission diagnosis versus age. **Table S4.** Socio demographic and clinical profile versus outcome of children admitted to PICU in Mekelle, North Ethiopia.

**Additional file 2: Annex 1.** Data collection format (questioner).

## Abbreviations

ARH: Ayder Referral Hospital
CNS: central nervous system
MOF: multiple-organ failure
ICU: intensive care unit
PICU: pediatric intensive care unit
WHO: World Health Organization
LOS: length of hospital stay
USA: United States of America
GCS: Glasgow coma scale
MV: mechanical ventilation
AFP: acute flaccid paralysis
AOR: adjusted odds ratio
COR: crude odds ratio
