# Supplementary material for: Assessment of pattern and treatment outcome of patients admitted to pediatric intensive care unit, Ayder Referral Hospital, Tigray, Ethiopia, 2015
Source: BMC Res Notes. 2018 May 24;11:339. doi: 10.1186/s13104-018-3432-4 (PMC5968617; doi:10.1186/s13104-018-3432-4)
Supplement: Supplementary file 1 — Additional file 1: Table S1. Patients’ outcome at the end of ICU stay. Table S2. Mortality across diagnostic categories (N = 34). Table S3. Admission diagnosis versus age. Table S4. Socio demographic and clinical profile versus outcome of children admitted to PICU in Mekelle, North Ethiopia. [file 13104_2018_3432_MOESM1_ESM.docx]

**Tables**

**Table S1**

| Variable | Number of patients | Percentage (%) |
| --- | --- | --- |
| 1. Patient outcome | | |
| - 1. Death | 34 | 8.5 |
| Female | 18 | 53 |
| Male | 16 | 47 |
| 1.2 ) Survival | 366 | 91.5 |
| 1.2.1Discharge | 65 | 16.3 |
| 1.2.2) Transfer to ward | 251 | 62.8 |
| 1 .2.3) Refer to other institution | 2 | 0.5 |
| 1.2.4) Went against | 48 | 12 |
| 1. Based on immediate cause of death | | |
| Respiratory arrest | 8 | 22.9 |
| MOF | 15 | 42.9 |
| Cardiac arrest | 12 | 34.3 |

**Table S2**

| Variable | Frequency | Percentage |
| --- | --- | --- |
| Diagnosis | | |
| Meningitis | 8 | 23.5 |
| Cardiogenic shock | 7 | 20.6 |
| CHF | 3 | 8.8 |
| Post-operative | 3 | 8.8 |
| Pneumonia | 3 | 8.8 |
| Others | 3 | 8.8 |
| Septic shock | 2 | 5.8 |
| AGN | 2 | 5.8 |
| TBI | 1 | 2.9 |
| Electrolyte imbalances | 1 | 2.9 |
| Others |  |  |

Others= Malignancy, poisoning, near-drowning, disseminated infection, brain tumor and alcohol intoxication

**Table S3.**

| Admission diagnosis | Less than five year  N (%) | Above five year  N (%) | Total patients admitted with same Dx N (%) | |
| --- | --- | --- | --- | --- |
| Asthma(HAAD) | 19 (73)* | 7 (27) | 26(6.5) | |
| DKA | 8 (26.6) | 22 (73.4)** | 30 (7.5) | |
| AGN | 28 (68.3)* | 13 (32.7) | 41 (10.3) | |
| CHF | 15 (41.6) | 21 (59.4) | 36 (9) | |
| Meningitis | 30 (68.2)* | 14 (32.8) | 44 (11) | |
| TBI | 4 (33.3) | 8 (67.7)** | 12(3) |  |
| Electrolyte imbalance | 10 (83.3)* | 2 (17.7) | 12 (3) |  |
| Croup | 25 (89.3)* | 3 (11.7) | 28 (7 ) |  |
| Septic shock | 9 (81.8)* | 2 (18.2) | 11 (2.8) |  |
| Cardiogenic shock | 0 | 8 (100)** | 8 (2) |  |
| Post-operative | 33 (76.6)* | 10 (23.4) | 43 (10.8) |  |
| Status-epilepticus | 9 (7.5) | 3 (25) | 12 (3) |  |
| UAO | 7 (58.3) | 5 (42.7) | 12 (2.5) |  |
| AFP | 0 | 100(3)** | 0.8(3) |  |
| Pneumonia | 90(18)* | 10(8) | 5(26) |  |
| Others (Malignancy, poisoning, near-drowning, brain tumor and alcohol intoxication ……16(64) | | | |  |

*common in under five children , **common above five children

**HAAD**-hyperactive air way disease, **DKA**-Diabetic ketoacidosis, **AFP**-acute flaccid paralysis

**CHF**-Congestive heart failure, **UAO-**upper air way obstruction, **AGN**-Acute glomerulonephritis

**Table S4**

| Variable | | Outcome | | | | COR (95%CI) | p-value | | AOR (95%CI) | |
| --- | --- | --- | --- | --- | --- | --- | --- | --- | --- | --- |
|  |  | Survived | | Dead | |  |  | |  | |
| **Age** |  | | |  | |  |  | |  | |
| Less than one year | | | 93(23.3%) | 8(2%) | | 0.62(.23-1.6) | 0.06 | | 0.17(.03—1.1) | |
| 1-2 year | | | 59(14.75%) | 3(0.75%) | | 0.36(.09-1.4) | 0.10 | | 0.2(.26-1.4) | |
| 2-5 year | | | 81(20.2%) | 6(1.5%) | | 0.53(.18-1.5) | 0.55 | | 0.6(.12-3.5) | |
| 5-11 year | | | 68(17%) | 8(2%) | | 0.85(.3-2.3) | 0.18 | | 0.27(.04-1.8) | |
| 11-18 year | | | 63(6.3%) | 9(2.25%) | | 1 |  | | 1 | |
| **Gender** | | |  |  | |  |  | |  | |
| Male | | | 197(91.6%) | 18(8.4%) | | 0.96(.47-1.9) | 0.95 | 0.97(0.32-2.8) | | |
| Female | | | 169(91.4%) | 16(8.6%) | | 1 |  | | 1 | |
| **Diagnosis** | | |  |  | |  |  | |  | |
| Meningitis | | | 36(81.8%) | 18.2) | | 4.5(1.1-18) | 0.86 | | 0.83(.09-7.3%) | |
| Septic shock | | | 9(94.5%) | 2(5.5%) | | 4.5(.66-30.8) | 0.08 | | .025(.001-0.9) | |
| Cardiogenic shock | | | 1(12.5%) | 7(87.5%) | | 14(12.9-15.6) | 0.04* | | 6.9(.8-52) | |
| Acute flaccid paralysis | | | 2(66.7%) | 1(33.3%) | | 10.2(.7-14.6) | 0.001* | | 47(11.1-19.5) | |
| Pneumonia | | | 17(85%) | 3(15%) | | 3.6(.67-19.4) | 0.23 | | 4.8(0.37-64) | |
| others | | | 61(95.3%) | 3(4.7%) | | 1 |  | | 1 | |
| **Infectious disease** | | |  |  | |  |  | |  | |
| No | | | 247(92.8%) | 19(7.1%) | | 1 |  | | 1 | |
| Yes | | | 119(88.8%) | 15(11.2%) | | 1.6(0.8-3.3) | 0.015* | | 5.7(1.7-14.4) | |
| **Comorbid illness** | | |  |  | |  |  | |  | |
| No | | | 210(96.7%) | 7(3.3%) | | 1 |  | | 1 | |
| Yes | | | 156 (85.2%) | 27 (14.8%) | | 5.2(2.2-12.2) | 0.002* | | 10.2(2.4-44) | |
| **Patient condition** | | |  | |  |  |  | |  | |
| Medical | | | 311(91.2%) | 30(8.8% | | 0.6(.17-2.1) | 0.6 | | 0.6(.05-6.5) | |
| Surgical | | | 36(97.3%) | 1(2.7%) | | 0.17(.17-1.8) | 0.4 | | 4.5(.1-18) | |
| Medical and surgical | | | 19(86.4%) | 3(13.6%) | | 1 |  | | 1 | |
| **Sources of admission** | | |  |  | |  |  | |  | |
| EOPD | | | 269(93.4%) | 19(6.6%) | | 1 |  | | 1 | |
| ROPD | | | 5(100%) | 0 | | 0.2(0.1-3) | 0.99 | | 0.5(.3-9.05) | |
| Transfer from ward | | | 52(80%) | 13(20%) | | 3.5(1.6—7.6) | 0.43 | | 0.56(.13-2.3) | |
| Transfer from recovery | | | 40(95.2%) | 2(4.8%) | | 0.7(.16-3.1) | 0.5 | | 0.1(0.5-14) | |
| **Length of ICU stay** | | |  |  | |  |  | |  | |
| Less than 2 days | | | 81(86.2%) | 13(13.8%) | | 1 |  | | 1 | |
| 2-7 days | | | 228(93.4%) | 16(6.6%) | | 0.43(.20-0.94) | 0.001* | | 0.09(.023-.39) | |
| 7-14 days | | | 40(93%) | 3(7%) | | 0.46(.12-1.7) | 0.02* | | 0.094(0.1-0.78) | |
| 14-28 days | | | 14(93.3%) | 1(6.7%) | | 0.44(.05-3.6) | 0.01* | | 0.019(.01-.45) | |
| >28 days | | | 3(75%) | 1(25%) | | 2(,2-21) | 0.53 | | 2.4(.14-39) | |
| **Need for MV** | | |  |  | |  |  | |  | |
| No | | | 356 (92.7%) | 28 (7.3%) | | 1 |  | | 1 | |
| Yes | | | 10 (62.5%) | 6 (37.5%) | | 7.6(2.6-22.5) | 0.007* | | 17.6(2.2-14) | |
| **Level of GCS** | | |  |  | |  |  | |  |  |
| less than 8 | | | 31(83.8%) | 6(16.2%) | | 2.5(.95-6.5) | 0.04* | | 7.74(1.1-54) | |
| 9-12 | | | 10(77%) | 3(23%) | | 3.9(1-15) | 0.29 | | 4.2(0.28-61.5) | |
| 13-15 | | | 325(92.9%) | 25(7.1%) | | 1 |  | | 1 | |
| **Need for inotropes** | | |  |  | |  |  |  | | |
| Yes | | | 349 (87.25) | 17 (12.7) | | 12.7(5.4-29.6) | 0.000* | | 10.4(3.7-29) | |
| No | | | 21 (5.25) | 13 (3.25) | | 1 | | | 1 | |

*=P<0.05

COR=Crude odds ratio ROPD-Regular out patient department

AOR=Adjusted odds ratio EOPD- Emergecy outpatient department

MV-mechanical ventilation

GCS-Glasgow coma scale
