# Supplementary material for: Assessment of pattern and treatment outcome of patients admitted to pediatric intensive care unit, Ayder Referral Hospital, Tigray, Ethiopia, 2015
Source: BMC Res Notes. 2018 May 24;11:339. doi: 10.1186/s13104-018-3432-4 (PMC5968617; doi:10.1186/s13104-018-3432-4)
Supplement: Supplementary file 2 — Additional file 2: Annex 1. Data collection format (questioner). [file 13104_2018_3432_MOESM2_ESM.docx]

**Annex I-Data collection format**

Current Socio-demographic and clinical Data

1-Age in month______________

- Age –in years

1. Less than one year

2.1-2 years

3.2-5 years

4.6-11 years

5.12-18 years

2-Sex

Male

Female

3-Admission Diagnosis (main reason for ICU admission)

1. Asthma 9. Croup

2. DKA 10. Septic shock

3. AGN 11. Cardiogenic shock

4. CHF 12. Post-operative patients

5. Meningitis 13. Upper air way obstruction

6. TBI 14. Acute flaccid paralysis

7. Electrolyte imbalances 15.Pneumonia with or without sepsis

8. Status Epileptics 16.Others (poisoning, malignancy, AKI, CKD, near drawing, UGIB…)

4- Infectious

1. Yes

0. No

5- If the answer of Q4 is yes how many system are affected

1. One 2.two 3.Three 4.More than three

1. Respiratory

2. Cardiac

3. CNS

4. GIT

5. Renal

6. Sepsis

7. Non specific

6- Noninfectious 1.Yes 0.No

7- If the answer for Q 4 is yes which system is affected?

A) Respiratory F)-Renal H)-Neoplasm

B)-Cardiac G)-Hematology I)-CTD

D)-GIT C)-CNS J)-Trauma

E)-Endocrine K)-Others_____

L)-Miscellaneous (poisoning, drawing...) ______

8- Comorbid illness (other additional diagnosis)

1. Yes

o. No

9-If answer for Q 8 is yes, number of comorbid illness?

1. One

2. Two

3. Three

4. More than three

1. HIV<Y> 7. Electrolyte imbalance<Y>

2. Meningitis<Y> 8. Pneumonia<Y>

3. TB<Y> 9. Post-operative patients<Y>

4. Anemia<Y> 10. Seizure<Y>

5. SAM<Y> 11. Aspiration pneumonia<Y>

6. AGE<Y> 12.Others<Y> (candidiasis, Skull fracture…)

10- Patient category

1. Medical

2. Surgical

3. Medical and surgical (both)

11-Admission sources

1 .EOPD

2. ROPD

3. Transfer from ward

4. Transfer from recovery

12- Total length of stay by days in ICU

-By number of days---

- Category

1. Less than 2 days

2.2-7 days

3.7-14 days

4.14-28 days

5. Greater than 28 days

13- Are they candidate for Mechanical ventilator during ICU stay?

1. Yes 0. No

14-If the answer of Q13 is yes were they put on Mechanical Ventilator?

1. Yes 0. No

15- If the answer is yes for how many days the patient stayed on MV?

-By number of days-----

-Category

1. Less than 1 day

2.2-7 days

3. Greater than 7 days

16-If the answer for Q14 is No, what is the reason?

1. Absence of MV

2. Other reason

3. Not documented

17-Mentation of the patient at admission (GCS)

1. Less than 8

2. 9-12

3. 13-15

18- Need for inotropes 1. Yes 0. No

19-Outcome of the patient

1. Death

2. Survive

20-If the answer of Q17 is survived, what is the patient condition?

1. Discharged with improvement

2. Discharge against medical advice

3. Transfer to ward

4. Referred to other institution

21-Cause of death

1. Respiratory failure

2. Cardiac arrest

3. MOF (multiple organ failure
